# Optimized Treatment of Interleukin (IL-1)-Mediated Autoinflammatory Diseases: Impact of Disease Activity-Based Treatment Adjustments

**DOI:** 10.3390/jcm13082319

**Published:** 2024-04-17

**Authors:** Tatjana Welzel, Beate Zapf, Jens Klotsche, Özlem Satirer, Susanne M. Benseler, Jasmin B. Kuemmerle-Deschner

**Affiliations:** 1 Pediatric Rheumatology, University Children’s Hospital Basel, University of Basel, 4031 Basel, Switzerland; 2 Pediatric Research Centre, University Children’s Hospital Basel, University of Basel, 4031 Basel, Switzerland; 3 Division of Pediatric Rheumatology, Department of Pediatrics, autoinflammatory reference centre Tuebingen, University Hospital Tuebingen, 72076 Tuebingen, Germany; 4 German Rheumatism Research Centre Berlin, 10117 Berlin, Germany; 5 Pediatric Rheumatology, Department of Paediatrics, Alberta Children’s Hospital, Cumming School of Medicine, University of Calgary, Calgary, AB T2N 4N1, Canada; 6 Children’s Health Ireland (CHI), D01 R5P3 Dublin, Ireland

**Keywords:** autoinflammatory disease, FMF, CAPS, TRAPS, remission, personalized medicine, effectiveness, exposure–response, outcome, monitoring

## Abstract

**Background:** Effective control of disease activity in Interleukin-1 autoinflammatory diseases (IL-1 AID) is crucial to prevent damage. The aim was to longitudinally analyze the impact of protocolized disease activity-based treatment adjustments in a real-life cohort. **Methods:** A single-center study of consecutive children with IL-1 AID followed between January 2016 and December 2019 was performed. Demographics, phenotypes, genotypes, inflammatory markers, physician (PGA), and patient/parent (PPGA) global assessment were captured. Disease activity and treatment changes were assessed. The impact of distinct parameters on disease activity trajectories was analyzed. **Results:** A total of 56 children were included, median follow-up was 2.1 years reflecting 361 visits. Familial Mediterranean Fever was the most common IL-1 AID. At the first visit, 68% of the patients had moderate/severe disease activity. Disease activity-based treatment adjustments were required in 28/56 children (50%). At last follow-up, 79% had a well-controlled disease. Both PGA and PPGA decreased significantly over time (*p* < 0.001; *p* < 0.017, respectively), however, both differed statistically at last visit (*p* < 0.001). Only PGA showed a significant estimated mean decrease across all IL-1 AID over time. **Conclusions:** Disease activity-based treatment adjustments can effectively refine treat-to-target strategies, enable personalized precision health approaches, and improve outcomes in children with IL-1 AID.

## 1. Introduction

Interleukin-1-mediated autoinflammatory diseases (IL-1 AID) are caused by pathogenic gene variants encoding the inflammasome, resulting in excessive release of (pro-) inflammatory cytokines [1]. Clinical characteristics of active IL-1 AID include recurrent fever, inflammation of the central nervous system, eyes, skin, serous membrane, and the musculoskeletal system, typically paired with elevated laboratory inflammatory parameters [2]. Although awareness has increased in the past years, several patients face a long journey until being diagnosed or treated [3,4]. Uncontrolled disease activity can result in organ damage, disability, reduced health-related quality of life (HRQoL), school and work absenteeism, and increased risk of mortality [5,6,7]. Furthermore, both the patient and the entire family carry a significant psychological burden. Therefore, effective treatment with the achievement of no or as mild as possible disease activity is essential after diagnosis is made.

The approval of effective treatment for IL-1 AID and treatment recommendations, along with the availability of disease activity monitoring parameters, such as the autoinflammatory disease activity index (AIDAI), autoinflammatory disease damage index (ADDI), the physician global assessment (PGA), and the patient/parent global assessment (PPGA), as well as specific laboratory inflammatory makers have significantly improved the patients’ management [8,9,10,11,12,13]. Based on the growing evidence of a relationship between disease activity, drug exposure, and treatment response in inflammatory diseases, the AID management should take personalized treatment approaches and disease activity-based treatment adjustments into account.

Disease activity-based treatment adjustments, also known as the treat-to-target (T2T) strategy, contain regular disease activity monitoring in a patient and disease activity-based treatment adjustments to achieve the desired target of no or mild disease activity [14]. T2T strategies were originally developed for optimized treatment in patients with rheumatoid arthritis and have been adopted for the management of several other chronic diseases [15].

Recently, T2T strategies for IL-1 AID were published [16,17,18]. However, longitudinal real-life data of disease activity-based treatment adjustments and their impact on IL-1 AID in childhood is scarce. In addition, data on disease activity parameters or the most promising composite measures after established treatment to monitor disease activity in IL-1 AID are needed.

Therefore, the objectives of this study were (i) to longitudinally assess the disease activity of children and adolescents with different IL-1 AID diagnoses, (ii) to analyze the individually performed disease activity-based treatment adjustments and their response, and (iii) to evaluate the impact of distinct parameters on disease activity trajectories/changes.

## 2. Materials and Methods

This was a single-center study assessing disease activity and disease activity-based treatment adjustments in children and adolescents with IL-1 AID between 1 January 2016 and 31 December 2019. Pediatric patients aged ≤18 years diagnosed with cryopyrin-associated periodic syndromes (CAPS), tumor necrosis factor-associated periodic syndrome (TRAPS), the mevalonate kinase deficiency (MKD), and the Familial Mediterranean Fever (FMF) were included if they fulfilled AID classification criteria [19], or in the case of FMF and CAPS, met either the classification and/or the diagnostic criteria [19,20,21]. Children and adolescents cared for less than one year or those with fewer than three routine visits during the study period were excluded. Data were captured in the designated, institutional web-based Arthritis and Rheumatism Database and Information System (ARDIS, ARDIS2, axaris software & systeme GmbH, 89160 Dornstadt, Germany). Data analysis was performed pseudonymized. Ethics approval was obtained from the ethics committee of Karl Eberhard University Tuebingen (050/2021BO2).

### 2.1. Patient Related Data

Demographic data included clinical AID diagnosis, sex, age at symptom onset, and diagnosis, as well as follow-up time. Furthermore, for those patients with genetic testing, the genotype was recorded. The genotypes were classified as pathogenic, likely pathogenic, variants of unknown significance (VUS), likely benign, or benign according to the American College of Medical Genetics and Genomics and Infevers [22,23,24]. In addition, the treatment of interest was recorded and included colchicine or one of the following biological Disease-Modifying AntiRheumatic Drugs (bDMARD): IL-1 inhibition, IL-6 inhibition, and/or TNF-α inhibition. In each visit, disease activity and information on the treatment regimen (absolute dose and body weight-based dose, frequency/administration interval, and admission route) were captured. Data collection was performed for each patient at the following visits: (i) the first visit in the study period, (ii) follow-up visits, defined as routine visits every 3 to 6 months during the study period, and (iii) the last study visit, defined as the last documented routine visit before study end.

### 2.2. Treatment Regimen and Definitions

The treatment regimen was compared intra-individually between the visits. Treatment escalations were categorized as (i) treatment start, (ii) treatment switch, (iii) dose increase, and (iv) administration frequency increase. Treatment start was defined as treatment initiation of colchicine or bDMARDs in a former treatment-naïve IL-1 AID patient. Treatment switch was defined as a switch from colchicine to a bDMARD, switch between different bDMARDs or an add-on drug, e.g., bDMARD treatment added on colchicine maintenance treatment between study visits. Dose increase was defined as an increase in absolute colchicine dose (mg) or an increase in mg/kg bDMARDs dosages in children between study visits. Administration frequency increase was defined as a shortening of the administration interval for bDMARDs between study visits, e.g., anakinra administration twice daily instead of once daily or canakinumab administration switching from eight weekly (q8w) to four weekly (q4w).

### 2.3. Definition of Disease Activity

In accordance with published studies, the physician global assessment (PGA) was used for the physician’s perspective recorded on a 10 cm visual analog scale (VAS), with 0 representing no and 10 maximum disease activity. The patient/parent perspective was captured through the patient/parent global assessment (PPGA) and was recorded on the 10 cm VAS, with 0 representing no and 10 maximum disease activity. Furthermore, inflammatory markers were captured, including C-reactive Protein (CRP) and Serum Amyloid A (SAA) [16,25,26,27]. Disease activity categories were: (i) mild, if PGA ≤ 2 cm plus CRP < 1.5 mg/dL and/or SAA levels < 30 mg/L; (ii) moderate, if PGA > 2–5 cm plus CRP 1.5 < 2.5 mg/dL and/or SAA ≥ 30 < 50 mg/L; and (iii) severe, if PGA > 5 cm plus CRP ≥ 2.5 mg/dL and/or SAA ≥ 50 mg/L. An increase in disease activity was defined as a switch of category from mild to moderate, mild to severe, or moderate to severe.

### 2.4. Outcome

The primary outcome was disease activity at last study visit. Secondary outcomes included the number of episodes with increased disease activity over time, the number and characteristics of performed treatment adjustments, applied dosing regimens, treatment responses, and disease activity trajectories/changes.

### 2.5. Analysis

Patients’ characteristics were summarized using descriptive statistics. Categorical variables were presented as numbers (%) and continuous variables were shown as mean (SD) or as median (interquartile range, IQR 25. and 75. percentile). The proportion of patients with mild disease activity at first and last study visit was compared using the McNemar sign rank test. The change in disease activity category was analyzed using generalized linear mixed models, adjusting the baseline value to the previous study visit. The significance level in this study was defined as *p* < 0.05. A paired *t*-test was used to examine whether there was a significant difference in the means of the PGA or PPGA. For the exploratory analysis of the influence of genotype on disease activity, pathogenic and likely pathogenic variants were combined. Mean PGA and PPGA over time in different IL-1 AID was estimated by non-parametric local polynomial approximation. The Statistical analyses were conducted by SPSS 28.0.1.1 (IBM Corporation, Statistics for Windows, 2021, Armonk, NY, USA) and STATA 12.1 (StataCorp. LLC., College Station, TX, USA).

## 3. Results

In total, 56 children and adolescents with IL-1 AID were included, with 19 (34%) being girls. The median age at symptom onset for the entire cohort was 2.5 years (IQR 0.5; 4.1). Children with CAPS showed the first disease symptoms early in infancy (median 0.3 years [IQR 0.2; 0.5]). The median age at diagnosis for the entire cohort was 4.9 years (IQR 3.0; 7.7). At the first study visit, the median age was 4.9 years (IQR 3.3; 8.1). FMF was the most common diagnosis, seen in 46/56 (82%) patients, followed by CAPS diagnosed in 9/56 (16%), and 1 child was diagnosed with TRAPS. Eight out of nine children with CAPS had a moderate and one had a mild phenotype. In 63% of the FMF patients, at least one (likely) pathogenic variant or one VUS was detected; the remaining FMF patients were diagnosed clinically. The median follow-up time for the whole cohort was 2.1 years (IQR 1.4; 2.7) corresponding to a total of 361 visits. At the first visit, 15/56 children (27%) had already received colchicine. Of these, 14 children were diagnosed with FMF and one with CAPS. Only one FMF patient had been started on a combination treatment of anakinra and colchicine at enrolment (Table 1 and Supplementary Tables S1 and S2).

### 3.1. Disease Activity over Time

**Figure 1:**
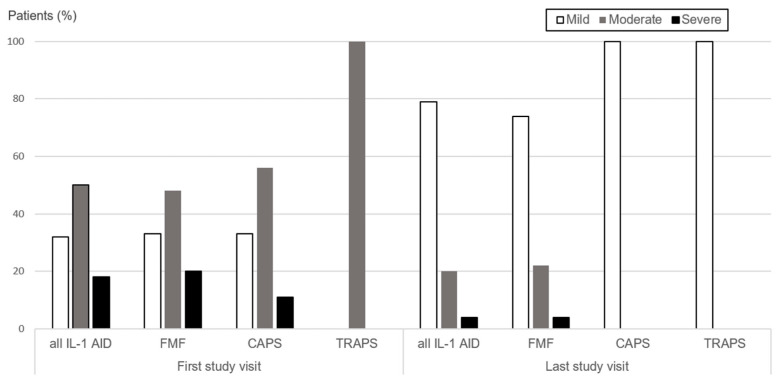
Disease activity in 56 children and adolescents with IL-1 AID at first and last study visit. Legend: Disease activity assessed at first and last study visit in children with IL-1 AID (all IL-1 AID; *n* = 56) and for IL-1 AID subgroups (FMF = 46, CAPS = 9, TRAPS = 1). Disease activity categories were defined as mild, moderate, and severe. A significant increase in mild disease activity category was shown between first and last study visit (McNemars sign rank test; all IL-1 AID *p* < 0.001, FMF *p* < 0.001, and CAPS *p* = 0.031). Abbreviations: IL: Interleukin, AID: Autoinflammatory disease, FMF: Familial Mediterranean Fever, CAPS: Cryopyrin-associated periodic syndromes, TRAPS: Tumor necrosis factor receptor-1-associated periodic syndrome.

At the first visit, 18/56 (32%) children presented with mild disease activity, 28 (50%) had moderate, and 10 (18%) severe disease activity. In contrast, at the last study visit, 44/56 (79%) children had mild disease activity, 10/56 (18%) moderate, and 2/56 (4%) severe disease activity. Over time, 36/56 (64%) children experienced at least one episode of increased disease activity. Changes were observed from mild to moderate in 17/36 (47%) children, from mild to severe in 12/36 (33%), and from moderate to severe in 7/36 (20%) children. A subsequent episode of increased disease activity was documented in 17/56 (30%), including mild to moderate in 13/17 (77%) and mild to severe in 4/17 (24%) (Figure 1 and Table 2).

### 3.2. Treatment Adjustments

#### 3.2.1. Disease Activity-Based Treatment Adjustments

The first episode of increased disease activity resulted in treatment adjustments in 28/36 (78%) children with disease activity increase. In 19/28 children, a new treatment was started, a treatment switch was performed in four and a dose increase was performed in eight children. Treatment adjustments were distributed similarly between groups of children diagnosed with FMF and CAPS. A subsequent episode of increased disease activity was seen in 17/56 (30%) children during follow-up. Treatment was adjusted in seven (41%) out of the 17 children. Adjustments included new treatment start in two children, switch in one, dose increase in three, and increase in administration frequency in one. CAPS patients experiencing a subsequent disease activity increase typically received a dose adjustment (Table 2).

#### 3.2.2. Specific Dosing Regimen

Median colchicine dose captured during the study for CAPS and FMF patients was 1 mg/day (maximum dose 1.5 mg/day) and 0.75 mg/day (maximum dose 1 mg/day), respectively. However, in those children with FMF carrying homozygous pathogenic/likely pathogenic *MEFV* variants, the median colchicine dose (1.13 mg/day) and the maximum colchicine dose (1.75 mg/day) were higher compared to FMF patients carrying heterozygous (likely) pathogenic variants (median dose 0.75 mg/day, maximum dose 1 mg/day). Four patients with FMF received anakinra; the median dose was 1.55 mg/kg once daily with a maximum dose of 1.62 mg/kg once daily. For canakinumab in CAPS, the median dose and the maximum dose was 3.93 mg/kg and 6.13 mg/kg, respectively. The administration frequency ranged from q4w (*n* = 17 visits) to q8w (*n* = 8 visits). The maximum canakinumab dose for the TRAPS patient was 2.78 mg/kg q4w. The two FMF patients treated with canakinumab had a median dose of 3.2 mg/kg and a maximum dose of 4.2 mg/kg q4w.

### 3.3. Impact on Disease Activity Trajectories

#### 3.3.1. PGA and PPGA

**Figure 2:**
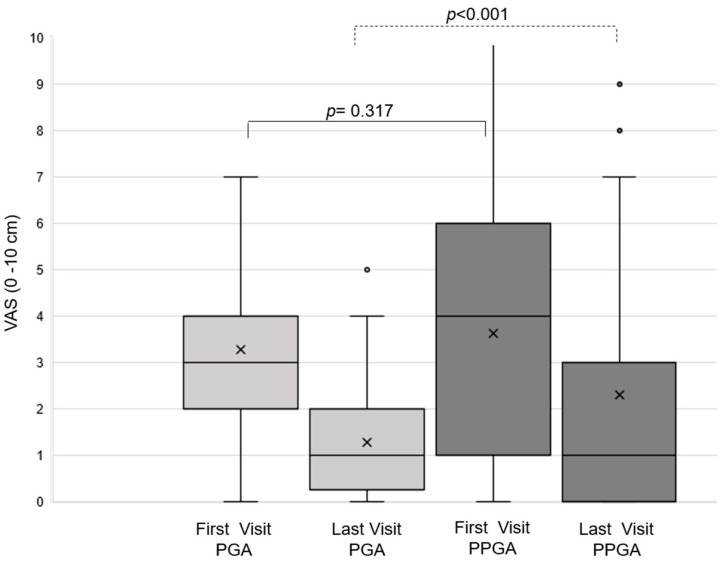
The association of treatment and disease activity-based treatment adjustments with the physician global assessment (PGA) and patient/parent global assessment (PPGA) at the first and last visit. Legend: Boxplot of the physician-derived global assessment (PGA) and the patient/parent-derived global assessment (PPGA) assessed with the visual analog scale (VAS) at the first and last visit. At the first visit, the median PGA was 3 (mean 3.1 ± 1.6) and the median PPGA was 4 (mean 3.6 ± 2.9). At the last visit, the median PGA was 1 (mean 1.2 ± 1.1) and the median PPGA was 1 (mean 2.2 ± 2.6). The paired t-test indicated statistical differences between the PPGA and PGA at the last visit. Legend: mean value (x), median (-).

**Figure 3:**
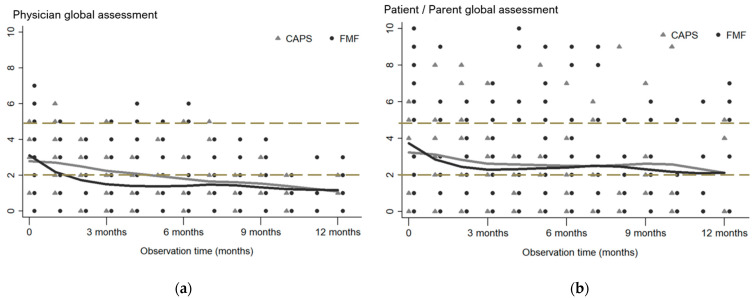
Longitudinal disease activity changes derived by the physician (PGA) and patient/parent (PPGA) in pediatric CAPS and FMF patients for 12 months after the first study visit. Legend: Course of (**a**) physician global assessment (PGA) and (**b**) patient/parent global assessment (PPGA) in children and adolescents with CAPS (grey triangle) and FMF (black dot) over the first 12 months after the first study visit estimated by local polynomial approximation. The PGA and PPGA are represented on the visual analog scale. Grey line: Local polynomial approximation for CAPS. Black line: Local polynomial approximation for FMF. Dashed gray line: Cut of PGA/PPGA > 2 cm and PGA > 5 cm. Abbreviations: FMF: Familial Mediterranean Fever, CAPS: Cryopyrin-associated periodic syndromes.

Overall, patient/parent-derived global assessments (PPGA) of disease activity tended to be higher than physician-derived assessment (PGA) values. In 48/56 patients, both the PGA and the PPGA were available at the first and the last study visit. At the first visit, PGA and PPGA were comparable (*p* = 0.317). Within the study period, both measures decreased significantly (*p* < 0.001 and *p* < 0.017, respectively). PGA and PPGA differed statistically significantly (*p* < 0.001) at the last study visit (Figure 2). CAPS and FMF patients showed comparable mean PGA and PPGA over the first 12 months when assessed by non-parametric regression analysis (Figure 3).

#### 3.3.2. Parameter Trajectories/Changes

The mean change of PGA, PPGA, CRP, and SAA adjusted for baseline values was determined over observation time in all three IL-1 AID subgroups. The decrease in PGA over time reached statistical significance in all subgroups with an estimated mean decrease of −0.12 (*p* < 0.001) for CAPS, −0.15 (*p* = 0.001) for FMF, and −0.11 (*p* < 0.001) for TRAPS. Additionally, a significant estimated mean decrease for the CRP (−0.05; *p* = 0.026) was detected in CAPS only. The six-monthly change in PGA adjusted for baseline values showed a significant estimated mean decrease, irrespective of pathogenic/likely pathogenic variants, VUS, or clinically diagnosed IL-1 AID (Table 3).

## 4. Discussion

This is the first comprehensive study analyzing the impact of disease activity-based treatment adjustments longitudinally in children and adolescents with different IL-1 AID in a real-life cohort. At the first study visit, 68% had moderate to severe disease activity, two-thirds (64%) experienced at least one flare episode, and one-third had subsequent episodes over the study’s duration. Overall, the approach of iterative disease activity-based treatment adjustments resulted in significant clinical improvements; most patients (79%) were asymptomatic and met the criteria for the mild disease activity category at the last study visit. Importantly, PGA and PPGA were similar at the first visit but differed significantly at the last follow-up (*p* < 0.001). The mean change of the PGA adjusted for baseline values over time revealed a significant decrease independent of genotype, clinical diagnosis, or IL-1 AID subgroup. These results highlight the importance of disease activity-based treatment adjustments and emphasize the need to further investigate the impact of distinct disease activity parameters.

Disease activity-based treatment adjustments can improve the outcome of patients with IL-1 AID including achieving remission/mild disease activity, improving HrQoL, and reducing the risk of organ damage. A rationale for disease activity-based treatment adjustments is the interplay of patient-related (e.g., age, living circumstances), disease-related (e.g., genotype, organ damage), and treatment-related (e.g., pharmacokinetics, side effects) factors [28,29,30]. The integration of these factors results in individual disease activity trajectories, as demonstrated in this study. In IL-1 AID, the genotype has been shown to influence the clinical phenotype, disease activity, and risk of organ damage [31,32,33,34,35]. External triggers including stress, lack of sleep, infections, and exercise as well as comorbidities can impact individual disease activity [36,37]. This complex interplay mandates the need for personalized treatment adjustments. Consequently, standardized disease activity monitoring and disease activity-based treatment adjustments are crucial to optimize care [14]. Drug exposure–response relationships have been reported for some inflammatory diseases in the past, indicating the need for higher drug exposure to control high disease activity [38,39]. This study emphasizes the drug exposure–response relationship across different IL-1 AID. The performed individual adjustments resulted in well-controlled disease in the majority of children (79%). In addition, a trend towards higher drug dosing in children with homozygous pathogenic/likely pathogenic *MEFV* gene variants compared to heterozygous carriers was observed. The exposure–response relationship in IL-1 AID aligns with the published data: patients with severe CAPS phenotypes needed higher drug exposure to control disease activity in contrast to mild phenotypes [10,40]; FMF patients with homozygous pathogenic gene variants (*M694V*, *M680I*) or compound heterozygous gene variants (*M694V/M680I*, *M694/V726A*) required higher colchicine doses (mean average range 1.19 mg/day to 1.09 mg/day) compared to those with any heterozygous genotype (average dose 0.81 mg/day) [41]. Based on the drug exposure–response relationship in IL-1 AID, T2T strategies can help to avoid drug underexposure and associated treatment failure. Additionally, they may guide treatment tapering decisions [17,28,42,43]. Individualized disease activity-based treatments and the application of T2T recommendations in IL-1 AID can, therefore, reduce the risk of suboptimal disease management, associated morbidity, and long-term organ damage [10,16,17,44]. Furthermore, it can avoid drug overexposure with increased risk of adverse events and higher drug costs than needed [43,45,46,47,48]. The drug exposure–response relationship and individual factors need to be considered as they help to further refine and optimize the management of children and adolescents with IL-1 AID.

Iterative, standardized disease activity monitoring over the disease course is crucial in IL-1 AID management. It is the cornerstone for T2T approaches. In the past, the development of valid disease activity assessment tools, standardized outcome measures, and HrQoL instruments in pediatric rheumatology have been important milestones for enabling optimal patient care [49]. The disease activity and disease burden are commonly assessed and quantified by the physician (e.g., PGA) and the patient/parent (e.g., PPGA), by HrQoL tools and missed school/working days, measurement of inflammatory markers, assessment of systemic and organ-specific signs of active disease, and evaluation of disease damage [10,18]. Important milestones for disease activity monitoring in IL-1 AID included the validation of the AIDAI and the development of the ADDI [8,9]. Furthermore, the identification of advanced inflammatory markers such as SAA, S100A8/A9, and S100A12 facilitated the recognition of subclinical inflammation [50,51,52]. In this study, we used a composite score for the assessment of disease activity, combining inflammatory markers and PGA. In addition, the PPGA was captured iteratively. The six-monthly mean changes in PGA, PPGA, SAA, and CRP adjusted for baseline values revealed a decrease in all disease activity parameters. Importantly, only PGA changes were found to be significant across all IL-1 subgroups. The estimated mean CRP decrease over time was only significant for CAPS. Mean PGA and PPGA values were similar at the first study visit; however, they differed significantly at the last visit. In line with the published evidence, patient/parent-derived values (PPGA) in this study tended to be higher than those of the physician (PGA) [53,54]. This might reflect the different concepts physicians and families consider when scoring. PPGA may also capture levels of fatigue, pain, psychological comorbidities, and impaired social participation experienced by the patient and family [55,56]. This highlights the importance of clarity on measuring constructs of disease activity, disease damage, burden of illness, and objective assessment of fatigue/sleep quality [57]. Recently, the *Protokolle in der Kinderrheumatologie* (PRO-KIND) initiative of the German Society for Pediatric Rheumatology (GKJR) has proposed a new composite tool for FMF [17]. This multidimensional instrument combines the assessment of flare frequency, missed school/working days, inflammatory markers, chronic sequelae/disease damage, the PGA, and the PPGA [17]. However, this tool has not been evaluated so far. Our results confirm that T2T approaches to IL-1 AID can effectively control disease activity and guide treatment adjustments. However, the overall burden of autoinflammation exceeds the concepts of disease activity and requires a multidimensional approach including capturing the disease impact on participation and mental health.

This study has several limitations. The sample size was small (*n* = 56); however, IL-1 AID are orphan diseases. The analysis was comprehensive, as the study population was well defined and the median follow-up of 2.1 years resulted in 361 visits. The study was conducted at a national reference center for patients with AID potentially biasing the sample towards children and adolescents with more severe, potentially difficult to treat disease courses. This may raise concerns about the limited generalizability of our results. However, the cohort included the entire disease severity spectrum across different IL-1 AID and analyzed the impact of disease activity-based treatment adjustments. Lastly, disease activity was assessed by commonly used composite scores, combining laboratory parameters and physician global assessments [16,25,26,27], neither AIDAI nor ADDI were formally integrated. However, both instruments are regularly reviewed at clinical routine visits, and, therefore, inform the global assessments.

## 5. Conclusions

This study demonstrates the importance of personalized disease activity-based treatment adjustments in IL-1 AID to optimize care and improve outcomes. It can help in preventing drug over- and underexposure. Prospective data collection and outcome assessments to further refine recently published T2T strategies and disease activity monitoring is critical. These insights will help to further optimize disease activity-based step-up but also step-down treatment in IL-1 AID.

## Figures and Tables

**Table 1:** Demographic and genetic characteristics of children with IL-1 AID.

	Total *n* = 56 (100%)	FMF *n* = 46 (82%)	CAPS *n* = 9 (16%)	TRAPS *n* = 1 (2%)
**Demographic characteristics**				
Female ^1^	19 (34)	17 (37)	2 (22)	0
Symptom onset, age in years ^2^	2.5 (0.5; 4.1)	2.9 (1.6; 4.7)	0.3 (0.2; 0.5)	2.7
Diagnosis, age in years ^2^	4.9 (3.0; 7.7)	5.1 (3.6; 7.5)	2.8 (1.9; 4.5)	12.9
First study visit, age in years ^2^	4.9 (3.3; 8.1)	5.1 (3.7; 8.0)	3.2 (1.9; 4.5)	13.0
Follow-up, years ^2^	2.1 (1.4; 2.7)	1.9 (1.4; 2.5)	2.5 (2.3; 2.9)	3.0
**Genetic variants**				
Pathogenic/likely pathogenic ^1^	28 (50)	25 (54)	2 (22)	1 (100)
VUS ^1^	11 (20)	4 (9)	7 (78)	0

^1^ *n* (%); ^2^ median (IQR). Abbreviations: *n*: Number of patients, IQR: Interquartile range, FMF: Familial Mediterranean Fever, CAPS: Cryopyrin-associated periodic syndromes, TRAPS: Tumor necrosis factor receptor-1-associated periodic syndrome, VUS: Variant of unknown significance.

**Table 2:** First and subsequent episodes of disease activity increase and performed treatment adjustments in children with IL-1 AID.

	Total *n* = 56	FMF *n* = 46	CAPS *n* = 9	TRAPS *n* = 1
**First episode of increased disease activity, *n* (%)**	36 (64)	30 (65)	5 (56)	1 (100)
Treatment adjustment in 28 out of 36 children *				
New treatment start, *n* (%)	19 (34)	15 (33)	3 (33)	1(100)
Treatment switch *n*, (%)	4 (7)	3 (7)	1 (11)	0 (0)
Dose increase *n*, (%)	8 (14)	7 (15)	1 (11)	0 (0)
Administration frequency increase, *n* (%)	0 (0)	0 (0)	0 (0)	0 (0)
**Subsequent episode of increased disease activity, *n* (%)**	17 (30)	13 (28)	4 (44)	0 (0)
Treatment adjustments in 7 out of 17 children *				
New treatment start, *n* (%)	2 (4)	2 (4)	0 (0)	0 (0)
Treatment switch, *n* (%)	1 (2)	1 (2)	0 (0)	0 (0)
Dose increase, *n* (%)	3 (5)	1 (2)	2 (22)	0 (0)
Administration frequency increase, *n* (%)	1 (2)	0 (0)	1 (11)	0 (0)

*n*: Number of patients, % percentage, * One patient might have received multiple treatment adjustments. Abbreviations FMF: Familial Mediterranean Fever, CAPS: Cryopyrin-associated periodic syndromes, TRAPS: Tumor necrosis factor receptor-1-associated periodic syndrome.

**Table 3:** Mean change of disease activity parameters adjusted for baseline values between IL-1 AID and genotypes.

**IL-1 AID Subgroups**									
	**CAPS**			**FMF**			**TRAPS**		
	**beta ^1^**	**95%CI**	***p* Value**	**beta ^1^**	**95%CI**	***p* Value**	**beta ^1^**	**95%CI**	***p* Value**
PGA	−0.12	−0.16; −0.08	<0.001	−0.15	−0.24; −0.06	0.001	−0.11	−0.17; −0.06	<0.001
PPGA	−0.09	−0.19; 0.02	0.100	−0.11	−0.32; 0.10	0.304	−0.08	−0.21; 0.05	0.247
CRP	−0.05	−0.10; −0.01	0.026	−0.10	−0.23; 0.03	0.130	−0.04	−0.09; 0.01	0.093
SAA	−2.49	−5.68; 0.71	0.127	−4.21	−10.62; 2.21	0.199	−2.26	−5.99; 1.47	0.236
**Genotypes**									
	**Pathogenic/likely pathogenic variants**			**VUS**			**No genetic testing/no VUS or (likely) pathogenic variants** **detected**		
	**beta ^1^**	**95%CI**	***p* value**	**beta ^1^**	**95%CI**	***p* value**	**beta ^1^**	**95%CI**	***p* value**
PGA	−0.13	−0.19; −0.08	<0.001	−0.09	−0.17; −0.01	0.020	−0.15	−0.26; −0.05	0.004
PPGA	−0.09	−0.23; 0.06	0.225	−0.08	−0.22; 0.06	0.280	−0.07	−0.28; 0.14	0.526
CRP	−0.07	−0.16; 0.01	0.073	−0.05	−0.12; 0.01	0.111	−0.04	−0.13; 0.05	0.444
SAA	−3.18	−8.97; 2.62	0.283	−2.67	−6.15; 0.80	0.132	−1.73	−10.25; 6.79	0.691

^1^ Changes per six months adjusted for values at first study visit. FMF: Familial Mediterranean Fever, CAPS: Cryopyrin-associated periodic syndromes, TRAPS: Tumor necrosis factor receptor-1-associated periodic syndrome, VUS: Variant of unknown significance, physician global assessment (PGA), patient/parent global assessment (PPGA), CRP: C-reactive Protein, SAA: Serum Amyloid A, CI: Confidence Interval.

## Data Availability

All relevant data are shown in the manuscript and Supplementary Material. Upon reasonable request and with obtained ethical approval, the dataset can be made available by the corresponding author.

## Supplementary Materials

The following supporting information can be downloaded at https://www.mdpi.com/article/10.3390/jcm13082319/s1, Supplementary Table S1: Detected gene variants. Supplementary Table S2: Treatment at first study visit in children with IL-1 AID.
