# Supplementary material for: Optimized Treatment of Interleukin (IL-1)-Mediated Autoinflammatory Diseases: Impact of Disease Activity-Based Treatment Adjustments
Source: J Clin Med. 2024 Apr 17;13(8):2319. doi: 10.3390/jcm13082319 (PMC11050771; doi:10.3390/jcm13082319)
Supplement: Supplementary file 1 [file jcm-13-02319-s001.zip › jcm-2943696-supplementary.pdf]

Supplementary material

Supplementary Table S1. Detected gene variants

	AMCG [22] classification	Variants	Heterozygous	Homozygous/ Compound heterozygous
**FMF (n=46)**	(Likely) Pathogenic, n=25	*M694V, V726A, M680Ile, K695N*	17	8
	VUS, n=4	*V722M, E148Q, A511V*	4	0
**CAPS (n=9)**	(Likely) Pathogenic, n=2	*E311K, D303N*	2	0
	VUS, n= 7	*Q703K, V198M*	7	0
**TRAPS (n=1)**	Pathogenic, n=1	*C55R*	1	0

*Homozygous: n=4, Compound heterozygous: n=4, n: Number of patients, FMF: Familial Mediterranean fever, CAPS: Cryopyrin-associated periodic syndrome, TRAPS: Tumor necrosis factor receptor-1-associated periodic syndrome, VUS: variant of unkown significance

Supplementary Table 2. Treatment at first study visit in children with IL-1 AID

Treatment	**Total** n=56	**FMF** n= 46	**CAPS** n= 9	**TRAPS** n=1
**Baseline visit, n (%)**				
Colchicine	15 (27)	14 (30)	1 (11)	0
Combination therapy	1 (2)	1 (2)		
- Anakinra plus colchicine	1 (2)	1 (2)	0	0

n: Number of patients, IQR: Interquartile range, FMF: Familial Mediterranean fever, CAPS: Cryopyrin-associated periodic syndrome, TRAPS: Tumor necrosis factor receptor-1-associated periodic syndrome, VUS: variant of unknown significance
